# Refractory Dermatophytosis and Oral Hairy Leukoplakia as Sentinel Manifestations of Advanced HIV Infection

**DOI:** 10.7759/cureus.99440

**Published:** 2025-12-17

**Authors:** Theodora Douvali, Varvara Vasalou, Stamatios Gregoriou, Alexander Stratigos, Electra Nicolaidou

**Affiliations:** 1 1st Department of Dermatology and Venereology, National and Kapodistrian University of Athens, "Andreas Syggros" Hospital for Skin and Venereal Diseases, Athens, GRC

**Keywords:** dermatophytosis, hiv infection, late hiv diagnosis, opportunistic fungal infection, oral hairy leukoplakia

## Abstract

Dermatophytosis is usually a benign superficial fungal infection, but in immunocompromised individuals, particularly those with advanced HIV infection, it may present with extensive, recurrent, and treatment-resistant lesions. We report the case of a 28-year-old woman with widespread dermatophytosis involving the trunk, extremities, and genital region, accompanied by oral hairy leukoplakia (OHL) on the lateral tongue. The lesions were refractory to repeated topical antifungal therapies, leading to diagnostic delay. Mycological analysis identified *Trichophyton mentagrophytes*. Further evaluation confirmed advanced HIV infection, with a cluster of differentiation 4 + (CD4+) T-cell count of 63 cells/mm³. Despite advanced immunosuppression, the HIV viral load was low-level and confirmed on repeat testing. The patient was treated with systemic terbinafine and antiretroviral therapy (ART) (Genvoya), resulting in marked improvement and the complete resolution of lesions within two months. This case emphasizes the diagnostic value of concurrent, treatment-resistant mucocutaneous manifestations as sentinel indicators of advanced HIV infection.

## Introduction

Dermatophytosis, commonly referred to as tinea or ringworm, is a frequent superficial fungal infection of keratinized tissues (skin, hair, and nails) caused primarily by species of *Trichophyton*, *Microsporum*, and *Epidermophyton* [1]. In most cases, these infections respond readily to topical or oral antifungal agents, depending on severity [1]. However, extensive, recurrent, or treatment-resistant disease is often associated with underlying immunosuppression. In individuals with advanced HIV infection, dermatophytosis may present with atypical morphology, multifocal or extensive involvement, and poor response to conventional therapy [2,3].

Among other mucocutaneous manifestations in HIV, oral hairy leukoplakia (OHL) is strongly linked to Epstein-Barr virus (EBV) infection and is widely recognized as an indicator of significant immunosuppression [4]. OHL typically appears as white or grey, non-removable, hairlike plaques, most commonly located on the lateral margins of the tongue but occasionally extending to other oral or oropharyngeal sites [5]. Although benign, its presence carries important diagnostic value. When treatment is indicated, management may involve topical agents such as gentian violet or retinoic acid or systemic antiviral therapy [6,7].

Late HIV diagnosis, defined as presentation with a cluster of differentiation 4 (CD4) count of <350 cells/μL or an AIDS-defining event, remains a major public health challenge [8]. It is associated with significant morbidity, premature mortality, and increased susceptibility to opportunistic infections [9]. Cutaneous manifestations, particularly those that are severe, recurrent, or refractory to standard therapies, can act as sentinel clinical markers that prompt the earlier identification of HIV infection [10].

Although dermatophytosis is frequently reported among people living with HIV, most studies provide limited clinical detail; data on extensive or treatment-refractory dermatophytosis remains sparse, especially when accompanied by mucosal manifestations such as OHL and unusual viro-immunological profiles [11].

Herein, we describe the case of a patient in whom refractory dermatophytosis and oral hairy leukoplakia acted as sentinel manifestations of advanced HIV infection, highlighting the diagnostic delay, challenges in antifungal management, and the broader public health implications for late HIV presentation in migrant populations.

## Case presentation

**Figure 1:**
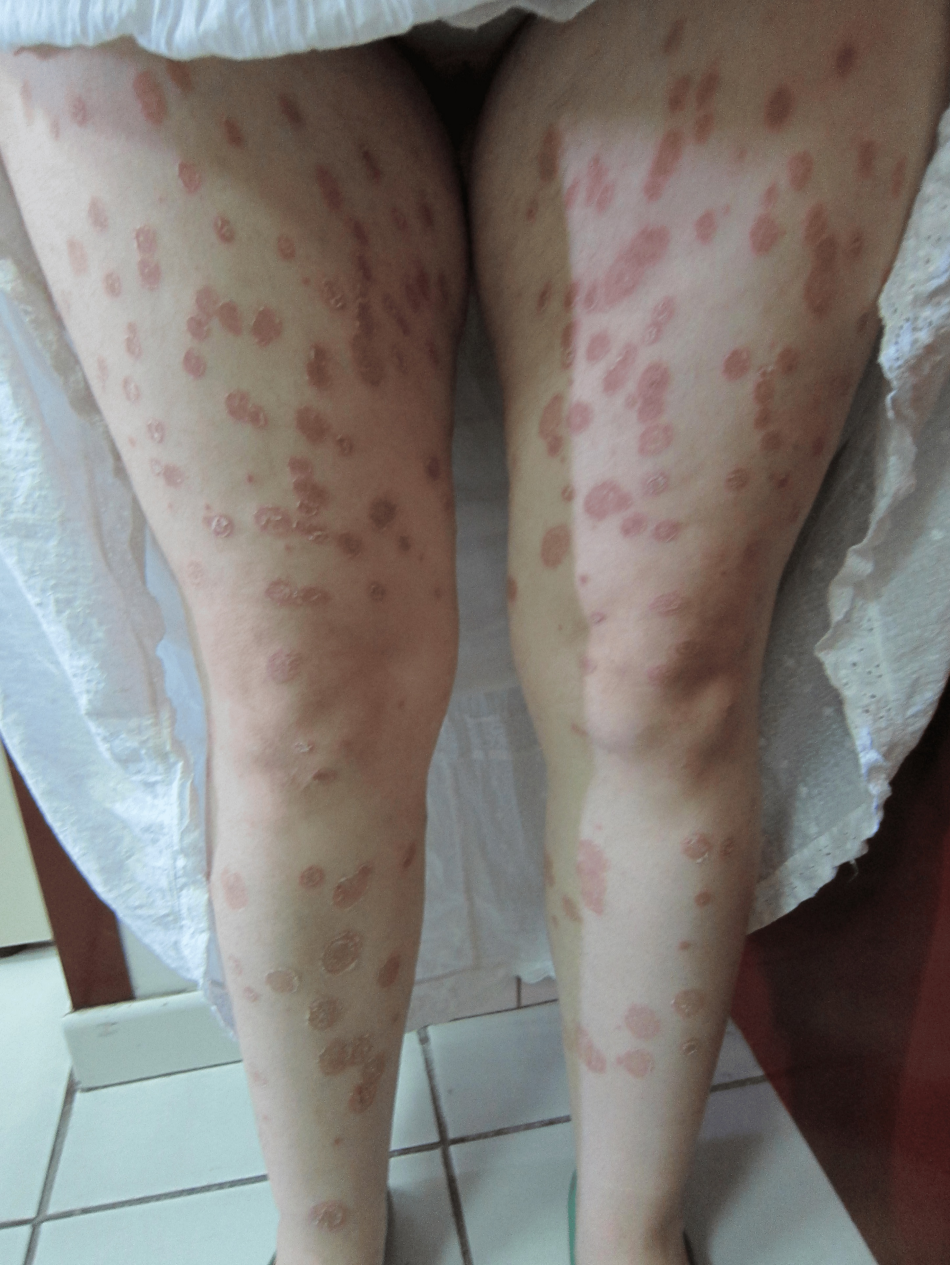
Extensive dermatophytosis of the lower extremities.

A 28-year-old woman of Albanian origin, residing in Greece, presented to the Infectious Diseases Unit at "Andreas Syggros" Hospital, a tertiary referral center for dermatological disorders in Athens, with extensive dermatophytosis affecting the trunk, extremities (Figure 1), and genital region, as well as OHL on the lateral tongue. The cutaneous lesions consisted of annular, erythematous, scaly plaques exhibiting centrifugal expansion with central clearing and active borders. The patient reported recurrent episodes over the preceding year. Her medical history included molluscum contagiosum, pediculosis, and herpes simplex virus infection. She also disclosed a history of sexual assault seven years earlier. These prior infections, while not severe, could have served as early clinical clues for underlying immunosuppression.

Previous treatments included multiple courses of topical antifungal agents with minimal or transient clinical response. No prior systemic antifungal therapy had been administered. There was no nail or scalp involvement, and the patient denied systemic symptoms such as fever, weight loss, or night sweats.

The diagnosis of dermatophytosis was established through clinical evaluation and laboratory confirmation. Direct microscopic examination of skin scrapings was performed using 10% potassium hydroxide (KOH) mounts and/or calcofluor white staining. Cultures were obtained on Sabouraud dextrose agar with chloramphenicol and actidione (cycloheximide) and incubated at 25°C-30°C. Mycological culture yielded *Trichophyton mentagrophytes*.

Given the recurrent and treatment-refractory nature of the infection and its inadequate response to standard therapy, an immunological and virological evaluation was undertaken. Immunological tests revealed no abnormalities. However, serological testing confirmed HIV infection via positive enzyme-linked immunosorbent assay (ELISA) and Western blot. The patient was also seropositive for anti-hepatitis B core antibody (HBc), anti-hepatitis B e-antibody (HBe), and anti-hepatitis B surface antibody (HBs), with negative hepatitis B surface antigen (HBsAg). Her CD4+ T-cell count was 63 cells/mm³. HIV viral load was 110 copies/mL, and low-level viremia was confirmed on repeat testing performed prior to the initiation of antiretroviral therapy (ART).

Treatment was initiated with systemic terbinafine at a dose of 250 mg daily for a total duration of eight weeks, alongside combination antiretroviral therapy (Genvoya). The patient exhibited significant clinical improvement, and the dermatophytic lesions resolved within two months of therapy.

## Discussion

This case underscores the importance of recognizing refractory mucocutaneous manifestations as indicators of advanced immunosuppression in individuals with HIV. While dermatophytosis is typically a benign and treatable superficial fungal infection, immunocompromised patients, particularly those with HIV, may present with severe, extensive, recurrent, or treatment-resistant disease [2,3]. The widespread, recurrent, and antifungal-resistant dermatophytosis observed in this patient aligns with reports describing severe presentations in individuals with advanced HIV infection, and in the present case, the repeated failure of topical therapy contributed to diagnostic delay [12].

OHL constituted an additional clinically significant finding in this case. Strongly associated with EBV, OHL is widely acknowledged as a marker of advanced HIV-related immunosuppression [4,5]. Although OHL is strongly associated with EBV and advanced HIV-related immunosuppression, it is important to distinguish it clinically from oral candidiasis, which is far more prevalent in patients with HIV infection [4,5]. Unlike candidiasis, OHL presents as non-removable white plaques typically on the lateral tongue and does not respond to antifungal therapy [6,7]. The coexistence of OHL with refractory dermatophytosis substantially increased clinical suspicion for HIV and prompted confirmatory testing. The markedly low CD4+ T-cell count at presentation confirmed advanced immunosuppression.

Late HIV diagnosis remains a major issue in both global and European contexts [13]. In 2023, over half (52.4%) of newly diagnosed individuals in the WHO European Region presented with advanced disease [14]. Such delays contribute to poorer outcomes, including heightened susceptibility to opportunistic infections, increased mortality, and ongoing transmission [8,9]. From a dermatological perspective, persistent or atypical cutaneous manifestations represent valuable diagnostic clues that can lead to the earlier detection of HIV infection [15]. This is particularly relevant in migrant or refugee populations, where access to healthcare and screening may be limited, potentially contributing to late presentation.

In this case, the combination of systemic antifungal therapy with antiretroviral treatment resulted in substantial improvement, demonstrating the crucial role of ART in restoring immune function and improving the control of opportunistic infections [9,10]. The recognition of refractory dermatophytosis and OHL as sentinel manifestations can prompt timely HIV testing and the early initiation of therapy, ultimately improving patient outcomes.

## Conclusions

This case highlights the need to consider underlying HIV infection in patients presenting with atypical, extensive, or treatment-resistant mucocutaneous disease. Refractory dermatophytosis and OHL should be regarded as potential sentinel markers of significant immunosuppression and warrant prompt HIV testing. Increased awareness among dermatologists and other frontline clinicians can facilitate earlier diagnosis, the timely initiation of antiretroviral therapy, and improved patient outcomes.
